# The Foolish Recoil from Registration

**Published:** 1920-04-17

**Authors:** 


### THE MATRONS' AND SISTERS' DEPARTMENT.

### THE FOOLISH RECOIL FROM REGISTRATION.

The plaintive murmur "What use is registration going to be to me?" is already beginning to make itself heard, and some nurses who joined in petitioning the Government to pass this necessary measure are not quite sure whether they intend to avail themselves of its protection now it is in being. The cause for this falling off in enthusiasm about State Registration is not far to seek. Clearly as nurses were warned that the State would not refuse to register and thus exclude from work nurses hitherto practising, although not up to the modern standard of training, it seems to have come as a surprise to many that the portal of registration will for a time be opened wide. Everyone can see that this course is wise and just—indeed, inevitable. But all the same, there are doubters who are persuading themselves that it is not to their own interest to "even themselves" with the *bona-fide* nurse by adding the prefix "registered" to their name.

Alas, the unity of the profession does not mean very much to women who have never seriously thought about these things, who have passed through their training-school without once learning that they had a duty towards other nurses no less than towards the public and the patient. For every broad-minded, open-hearted woman concerned about the honour of nursing we must expect to find another whose sole concern is with her own career. It is of little avail, then, to insist upon abstract consideration of public utility. The great bulk of nurses whose names ought to be inscribed upon the Register must be convinced that it will be for their own individual interest to take this step, or they will not trouble.

It is difficult for nurses to realise while they are in training, or have recently qualified, what a purely local reputation the certificate they prize so highly can probably boast. The public, even that part of it which employs nurses in institutions, is curiously ignorant of any but the hospitals in its immediate neighbourhood. There are a dozen hospitals in London and elsewhere whose certificate carries weight all the world over. But what Southerner knows the value of the training in a Midland or North country hospital. What North countryman has any faith in the training given in institutions south, east, or west? People simply do not know the names of the hospitals or of the doctors who lie outside their immediate district. The nurse who trusts for recognition solely to her certificate now registration has leaped into the arena will find herself for ever hampered in changing her locality. Always there will be a difficulty in explaining why she is not a registered nurse, and as time goes on this difficulty will become increasingly serious. The impression will gain ground that the only reason for not being registered is the existence of something irregular.

Years ago, when registration was being urged upon a profession which almost unanimously recoiled from it, the position was widely different. Had a hundred of the leading hospitals in those days advised their nurses against being registered, the measure must have become a dead-letter. But circumstances have changed. Not only are the training-schools enthusiastically welcoming the advent of State Registration, but a new factor has entered into the profession in the appointment of the Minister of Health. State Registration is a Government measure, not a mere party move, and assuredly the Minister of Health does not intend it to be a dead-letter. The whole outlook of nursing has been altered to an inconceivable extent by the Ministry, which is gradually taking over the health services of the country and gathering all nurses under its wing as servants of the State.

What opening will there be for unregistered nurses when every institutional or municipal post of any importance carries the stipulation that the applicant be a registered nurse, when every nursing home is under inspection and compelled to employ none but the registered, when all reputable employment agencies restrict their operations to the registered? From the merely selfish, self-interested point of view there will be no escape from State Registration. The corners of the nursing world which remain lurking-places for the unregistered will be cumbered with incompetents who have blundered over their training, or whose characters in the past will not bear the close scrutiny of the registration authorities.

As for some nurses on the register being better trained than others, is it not clear that this must always be the case? Registration is not intended to equalise. It aims at uniting. Assuredly not all the doctors on the register are equal. But registration has secured a unity without which their profession, like that of nursing, lacked cohesion and strength.

